# Comparison of the Burden Evolution of the Family Caregivers for Patients With Cancer and Nononcological Diseases Who Need Palliative Care: A Prospective Longitudinal Study

**DOI:** 10.1089/pmr.2022.0067

**Published:** 2023-07-17

**Authors:** Rodica Sorina Pop, Daniela Viorica Mosoiu, Aida Puia, Diana Tint

**Affiliations:** ^1^ “Iuliu Hatieganu” University of Medicine and Pharmacy, Cluj-Napoca, Romania.; ^2^ Transilvania University, Brasov, Romania.

**Keywords:** burden, family caregiver, nononcological disease, palliative care

## Abstract

**Background:**

The family caregiver (FCG) is with the patient from diagnosis till the end of life. The accumulated burden has a negative impact on the caregiver's quality of life and on his physical and emotional well-being.

**Objective:**

To quantify the burden of care for a patient with palliative needs, and to compare the burden experienced by caregivers for nononcological patients with those for cancer patients.

**Design:**

Prospective longitudinal study.

**Setting/Participants:**

One hundred forty patient–primary caregiver pairs participated in the study, which were separated into two groups: those who cared for patients with nononcological diseases (*n* = 63) and those who cared for patients with cancer (*n* = 77).

**Measurements:**

The burden measurement was assessed with Burden Scale for FCGs.

**Results:**

The average score of the FCG's burden was significantly higher in the nononcological group (45 ± 14.45 vs. 36.52 ± 15.05; *p* = 0.001). In the case of caregivers for cancer patients it is noticed that the caregivers' burden decreases after the intervention of the specialized team (45.58 ± 14.11 at T1 vs. 36.65 ± 16.10 at T2; *p* = 0.001). The burden values for caring for patients with nononcological diseases remained in the plateau, indicating incremental caregiver adaptation, although the rising trend is still present toward the end of the term (47.43 ± 13.32 vs. 56.69 ± 15.44; *p* < 0.001).

**Conclusions:**

The burden dynamics are different depending on the patient's disease, duration of care, degree of dependence, number of comorbidities, and on the intervention of the palliative care team that ensures the support of the caregiver for the palliative patient.

## Introduction

Patients who require palliative care due to a life-threatening illness go through multifaceted suffering, including physical, psychological, emotional, social, and spiritual pain. The family caregiver (FCG) is typically a person who is close to the patient, actively participates in the care process, and is not compensated. The caregiver is frequently a member of the patient's immediate family (spouse, son or daughter, or close friend or neighbor).^1^ He makes the connection between the patient and the multidisciplinary team, ensuring the continuity of care. Thus, he has a dual role, being at the same time, a supplier and beneficiary of the palliative care.^2,3^

The FCG follows together with the patient the trajectory of the disease that differs depending on the pathology. The patient with oncological disease, in the last months of life, shows the constant decline of the clinical state compared with the patient with organ failure who records a sinuous decline with numerous exacerbations and remissions but always the return being at a lower level of functioning.^4–6^ Another category of nononcological patients are the frailty elderly and patients with dementia whose decline of the general state spans several years.^4,5^

Pain, digestive symptoms, psychoemotional symptoms, and fatigue are more frequent in nononcological diseases than in cancer, but most of the time they are unrecognized or insufficiently reported.^7^ As the disease progresses, the primary caregiver's responsibilities are multiple,^8,9^ this one being in the position to manage the patient's care facing the challenges to maintain his daily normality.^10^

The burden of care represents the negative and multidimensional perception that the FCG perceives during the care of a person in palliative care.^11^ The FCG is not trained in what the medical care is concerned and the decision connected to the medication management,^12^ this increasing the uncertainty of his interventions and the burden felt,^13^ and decreases the quality of life.^14^ The paternalistic attitude embodied in decision making at different moments of the care,^15^ mainly in the care at the end of the patient's life^16^ as well as the management of conflicts with the other members of the family exacerbates the burden felt.^8^

## Aim

The study aimed at measuring and comparing the burden of caregivers for patients with cancer and noncancer diseases in palliative care stage, over three months of follow-up.

We present the following article in accordance with the STROBE reporting checklist.

## Materials and Methods

### Design and sample

This study was a prospective longitudinal one that assessed the burden of care for patients with palliative needs between two groups: a group of FCGs for patients with noncancer diseases (FCG1) and a group of FCGs for patients with cancer (FCG2). The study was conducted between February 1, 2019 and January 31, 2020, and included all patients who presented themselves to the Palliative Care Department and met the inclusion criteria.

The patients included in the study were >18 years old, Romanian language speakers, and met the criteria for palliative care according to the Regulation on the organization, operation, and authorization of palliative care service in Romania, patients with chronic progressive diseases and oncological patients in stages III or IV of disease.^17^ Each patient appointed his FCG, and in the case of patients with cognitive disorders, the person who accompanied the patient to the first consultation was co-opted in the study.

The inclusion criteria for the primary caregiver were as follows: only adults >18 years, who had not received palliative care yet, native fluent Romanian speakers, persons who did not receive remuneration for the service they provided, and people who did not have any conditions that would have impaired their cognitive function. The subjects included in the study reported no diagnosis whatsoever of cognitive impairment, and the Mini Mental State Examination (MMSE) test had a value between 28 and 30. All the aforementioned criteria were mandatory. Both the patient and the FCG were informed about the purpose and methodology of the study and filled in their written consent.

At the first presentation that coincides with the initiation of the palliative care, the FCG filled in a questionnaire with demographic data and the burden assessment questionnaire. This moment of caregiver's evaluation was named T0 and corresponds to initial evaluation. This first value represents the burden accumulated before the intervention of the palliative care team. The caregiver's characteristic questionnaire contains data such as age, gender, degree of kinship to the patient, living environment, living space, level of education, occupation, monthly average income per family member, number of family members, the number of hours spent caring for the patient, number of days absent from work, and quitting the job.

The patient's characteristic questionnaire contains age, gender, living environment, occupation, level of education, marital status, number of family member, and monthly average income per family member.

The follow-up visits have been made monthly for three months (T1–T3).

### Instrument used

The Burden Scale for Family Caregivers (BSFC) is a 28-item tool that assesses the burden of care for a patient in palliative care. It is an instrument that evaluates three domains of the FCGs burden: physical, emotional, and social. The total score for the 28 items of BSFC can be between 0 and 84. Each item is a statement in which the patient chooses the variant that suits his condition (they totally agree, they agree, they partially agree, and they do not agree) and each is quantified with a score of 0 to 3 points. The higher the value means the greater the burden of care. The increased internal consistency indicated by the Cronbach's alpha index of 0.92 was confirmed for the scale as a whole.^18^ The scale has increased reliability in assessing the total subjective burden, so the score obtained indicates its existence and severity.^18^ BSFC is a validated and available instrument in different languages, including Romanian, which allowed the assessment of the burden of the FCG's emotional and physical health.^19^

### Statistical analysis

The software package IBM SPSS v 26.0 for Windows was used to perform the statistical analysis of the data. Numeric variables are presented as mean value ± standard deviation (SD). The chi-squared test was used to compare the frequency of nominal variables. To evaluate the BSFC values both between the two samples and at different points in time we used the *t* test for independent samples. The threshold of statistical significance is considered *p* < 0.05.

### Ethical considerations

The research was approved by the Ethics Commission of the Transylvania University of Brasov and the Ethics Commission of the hospital. Eligible patients and their FCGs were informed of the purpose of the study and signed the informed and confidentiality consent form.

## Results

**FIG. 1.**
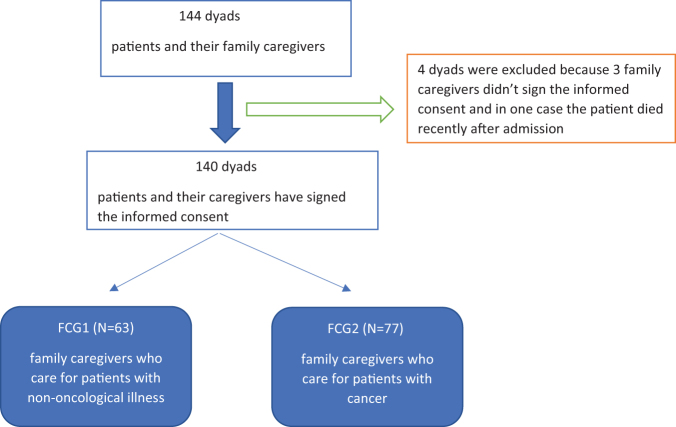
Algorithm for enrolling the dyads (patient and caregiver) in the study. FCG, family caregiver.

During the study, 144 patients and their FCGs were present for consultation or admission to the medical unit. Out of these, only 140 primary caregivers met the eligibility criteria and expressed written informed consent. The response rate was 97.22%. FCGs and patients included in the study were divided into two groups: 63 FCGs who care the patients with nononcological diseases (FCG1) and 77 FCGs who care patients with cancer (FCG2) as shown in Figure 1.

The characteristics of the patients are shown in Table 1. Noncancer patients are significantly older than those with cancer (78.38 ± 9.98 years vs. 72.32 ± 11.90 years; *p* = 0.001), have significantly higher number of comorbidities (42.86% vs. 33.77% with at least five chronic illnesses; *p* = 0.04), and almost all of them are completely reliant on palliative care, according to the Barthel score (96.83% vs. 72.73% patients with cancer; *p* < 0.00001).

**Table 1.** Characteristics of Patients Included in Study

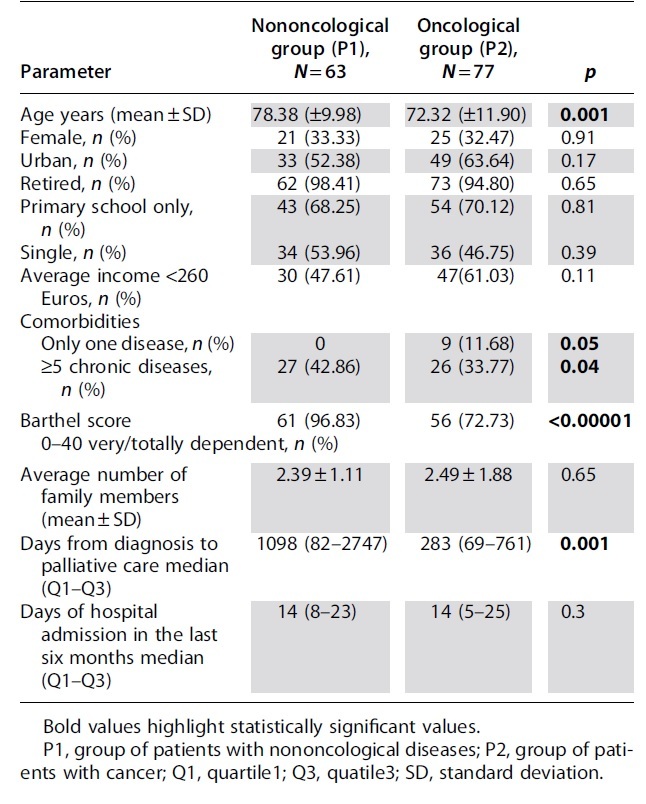

The median time interval between diagnosis and T0 (first presentation to palliative care services) is significantly much higher in the case of nononcological patients than in cancer patients (1098 days vs. 283 days; *p* = 0.001)

The most common oncological pathologies were as follows: digestive tract cancer (25%), bronchopulmonary cancer (14%), and breast cancer (13%). In descending order, the other locations of the malignant tumors were the following: liver and bile ducts (8%), gynecologic (7%), ear, nose and throat cancer (6%), brain (5%), pancreas (5%), and prostate (4%).

The most common nononcological disease was dementia (64% of all admissions), followed by stroke (27%) and congestive heart failure class New York Heart Association (NYHA) IV (6%).

There were no statistically significant differences between the two groups, according to the examination of the FCGs' characteristics (Table 2).

**Table 2.** Characteristics of the Family Caregivers

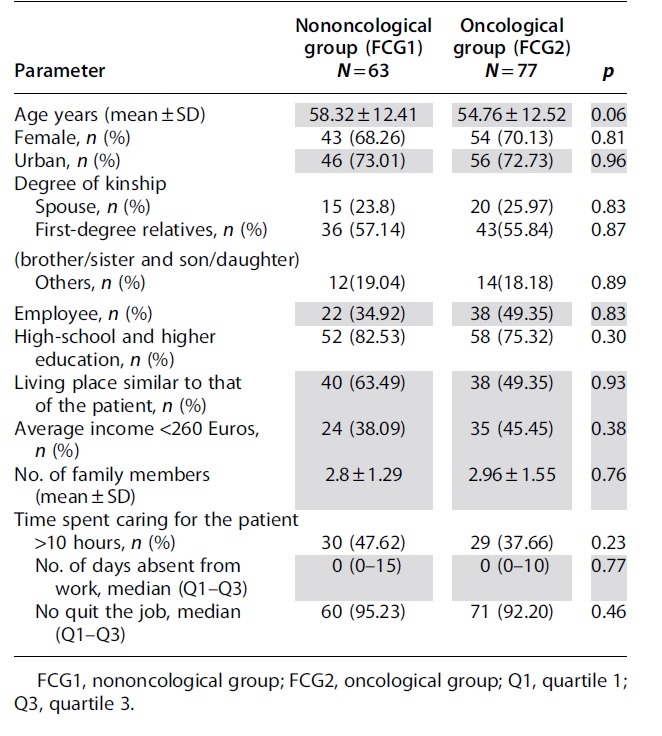

The two subgroups of FCGs do not significantly differ from each other. In more than half of the situations the patient's care is taken over by patient's son/daughter and in a quarter of the cases by the life partner. Although half of them give >10 hours a day for patient's care, absenteeism is negligible, and the vast majority do not give up their jobs.

The assessment of the care burden for the caregivers of the two groups got the values mentioned in Table 3.

**Table 3.** Burden Scale for Family Caregivers Score for Caregivers from the Two Groups

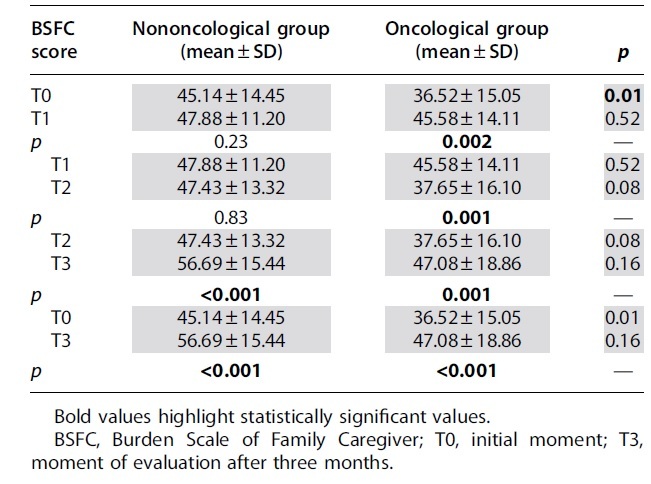

The examination of the average score reveals that at the time palliative care began, the nononcological group's caregivers had a much heavier burden than the oncological group's (45.14 ± 14.45 vs. 36.52 ± 15.05; *p* = 0.01). Throughout the three-month assessment, the burden score maintains its growing trend, but the differences between the oncological and nononcological group do not differ significantly.

**FIG. 2.**
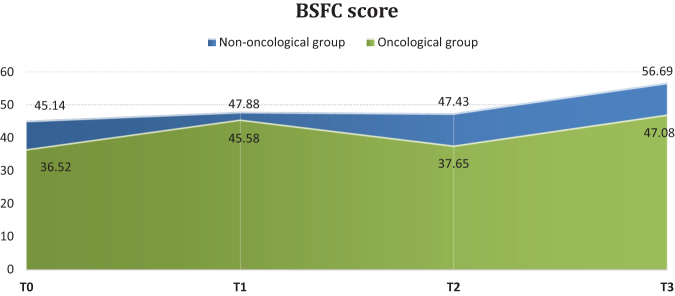
The average burden of family caregiver. BSFC, Burden Scale for Family Caregiver; T0, initial time; T1, evaluation at a month; T2, evaluation at two months; T3, evaluation at three months.

The score at moment T0 represents the burden value before the action of the palliative care team. This shows a low to moderate level of demand in the case of care for a patient with cancer, and a moderate level in the case of care for a patient with nononcological disease. At the end of the period (T3), a statistically significant increase is observed in both groups passing over the threshold of the severe degree of request especially in the group of those who care for nononcological patients (56.69 ± 15.44 in FCG1 vs. 47.08 ± 18.86 in FCG2; *p* = 016) as shown in Figure 2.

**FIG. 3.**
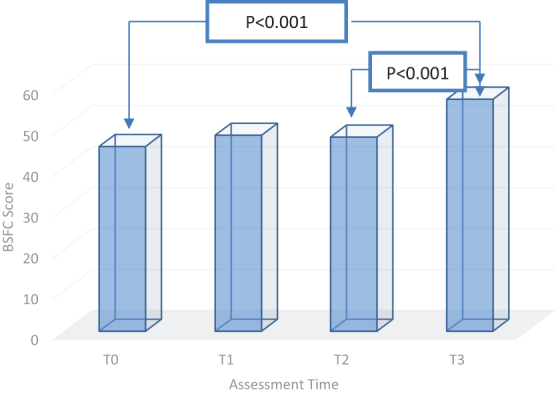
Burden dynamics of the caregivers for patients with nononcological disease.

The dynamics of the burden score differs between the two groups. The moment the palliative care was initiated, the caregivers for the patients with nononcological disease experience an increased degree of burden explained by the much longer period of domicile care and by numerous needs of the patient who is totally dependent. According to the results, in the first two months of evaluation, the burden remains almost flat, and increases significantly toward the end of the period (47.43 ± 13.32 vs. 56.69 ± 15.44 at T3; *p* < 0.001). The initial value of the burden is at the moderate level, the average value increasing statistically significant at the end of the three months, placing the FCG at a very high level with increased risk of psychosomatic disorders (45.14 ± 14.45 at T0 vs. 56.69 ± 15.44 at T3; *p* < 0.001) (Fig. 3).

**FIG. 4.**
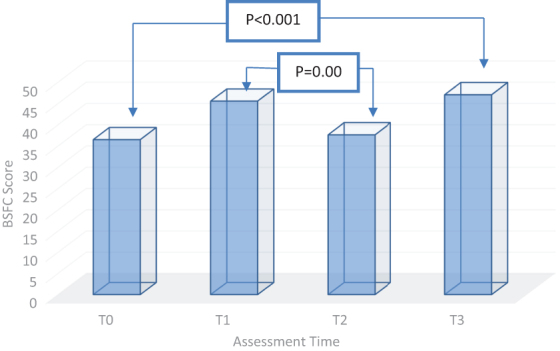
Burden dynamics of the caregivers for cancer patients.

Caregivers of patients with cancer, however, experienced a different load dynamic. The patients ended up being taken into palliative care earlier, with a caregiver's burden significantly lighter compared with that of the nononcological ones (36.52 ± 15.05 vs. 45.14 ± 14.45; *p* = 0.01). As the disease progresses, different symptoms appeared, out of which the pain of varying intensity was the most common. Thus, the caregivers experimented a burden that statistically significantly increases from one month to another. We observed a statistically significant reduction in the care load from time T1 to time T2 (45.58 ± 14.11 at T1 vs. 36.65 ± 16.10 at T2; *p* = 0.001), which could be attributed to the benefits of palliative care in achieving effective symptom control (Fig. 4).

Within the oncological group (P2), the burden of care is statistically significantly higher after three months since the first evaluation (36.52 ± 15.05 at T0 vs. 47.08 ± 18.86 at T3; *p* < 0.001), as shown in Figure 4.

## Discussions

The FCG's burden is a multidimensional notion in close relation with the care needs that increase as the disease progresses. The nononcological patient's care differs significantly compared with the care for a patient with cancer, mainly being influenced by the 3.8 times higher duration from diagnosis to the initiation of the palliative care. Within this period the whole care is assumed by the person involved in the patient's care.

The total dependence on a caregiver, and the large number of associated comorbidities represented other two factors that made nononcological patient's care much more laborious and burdensome.

Nononcological patients are statistically significantly older than cancer patients. Accumulated comorbidities have led to complications that increase care needs and increase their dependence for primary caregiver. From diagnosis to admission in palliative care department, the patient is at home, so all responsibilities are on the caregiver's shoulders, which makes the burden significantly higher than in cancer patients.

More than two thirds of the caregivers in both groups were female, making them more susceptible to decompensation from the stress of providing care.^20,21^ In half of the cases, the patient's care was taken over by the patient's son or daughter and in a quarter of the situations the patient's care was made by the life partner. They ultimately ended up putting the patient's needs ahead of their own and neglecting their own needs, which resulted in the development of clear signs of burden, such as anxiety-type psychoemotional disorders, depression, insomnia,^22,23^ as well as physical symptoms that reduced the patient's quality of life.^14^

The care of the patient with nononcological disease requires a different dynamic of the burden, showing a copping of the burden at a moderate value due to the capacity to gradually adapt over a longer care period. Toward the end of the period, the burden significantly increases reaching a high degree of request by exhausting their own adaptation reserves.

In the case of a cancer patient, the disease's progression is heterogeneous, with evident symptoms, greater mental distress after the diagnosis, uncertainty regarding the disease's course, and treatment adjustments that may have adverse effects. All of this requires earlier palliative care intervention to guarantee good symptom control. This affects the caregiver's workload, which significantly lessens when the patient's clinical state gets better. However, the tendency keeps going up in the last period, reaching a moderately high to high level of demand.

One of the care burden consequences is the occurrence of the caregiver's physical exhaustion, which leads to the low efficiency of his interventions and to the subjective perception of the worsening of one's own state of health.^3,24^ On the psychoemotional level, the burden leads to the accentuation of anxiety, depression with the increase of the feeling of uncertainty on their own actions and on the future.^23^

More than 40% of caregivers spend >10 hours a day taking care of the patients, neglecting the other family members and even themselves.

Early palliative care intervention has been shown to be effective in improving patient quality of life, lowering depressive episodes, and even increasing survival.^25^ In addition, identifying symptoms and managing them had the impact of lessening the FCG's burden (as another study also shown), by a significant decrease in workload after the intervention of the expert team.^25^

The FCG's load level and risk factors that make him susceptible to psychosomatic decompensation brought on by the difficulties of the assumed activities will be determined by an early evaluation of the caregiver.^26,27^ This can be explored further in the research on two levels: “prophylaxis” through the identification of risk factors and “curative” through the determination of the degree of demand for therapeutic action to lessen caregiver load.

The degree of the burden experienced by the FCG is revealed by a direct assessment of their overload. The context of the family, the number of family members involved, the domestic relationship, one's own health and/or the presence of certain diseases (particularly psychoemotional ones), as well as one's own perception of cultural norms and values are all factors that indirectly affect how much of a burden one perceives.^3,14,27^

### Limitations

The study limitations include the small number of subjects at baseline and an important reduction of several subjects at three months due to the patient's death. It also reflects a single center experience.

## Conclusions

Owing to the significantly longer period of care and greater dependence brought on by total incapacity, the FCG has a heavier burden when caring for nononcological patients than when caring for cancer patients.

The dynamics of caregiver burden are different, with bigger oscillations in the oncological group where symptom control benefits caregiver burden reduction.

In both categories, the burden grows as the illness worsens, and the caregiver's chance of developing psychosomatic illnesses rises as the ailment is neglected.

## Data Availability

This dataset is subject to ethical restrictions and data protection regulations that do not allow publication of data. All relevant data for the conclusions are presented in this article.

## Abbreviations Used

BSFC: Burden Scale for Family Caregivers
FCG: Family Caregiver
P: patient group
SD: standard deviation
